# First person – Savant Thakur

**DOI:** 10.1242/bio.054593

**Published:** 2020-07-24

## Abstract

First Person is a series of interviews with the first authors of a selection of papers published in Biology Open. Savant Thakur is first author on ‘HSP70 drives myoblast fusion during C2C12 myogenic differentiation’, published in BiO. Savant was a Ph.D. student in the lab of Professor Gordon S. Lynch at the Centre for Muscle Research, Department of Physiology, The University of Melbourne, working towards understanding the mechanisms of defective muscle repair in muscle diseases and wasting disorders. Sadly, Savant passed away on June 16, 2019 and so one of his supervisors, Professor Lynch, spoke to BiO about Savant's work and character.


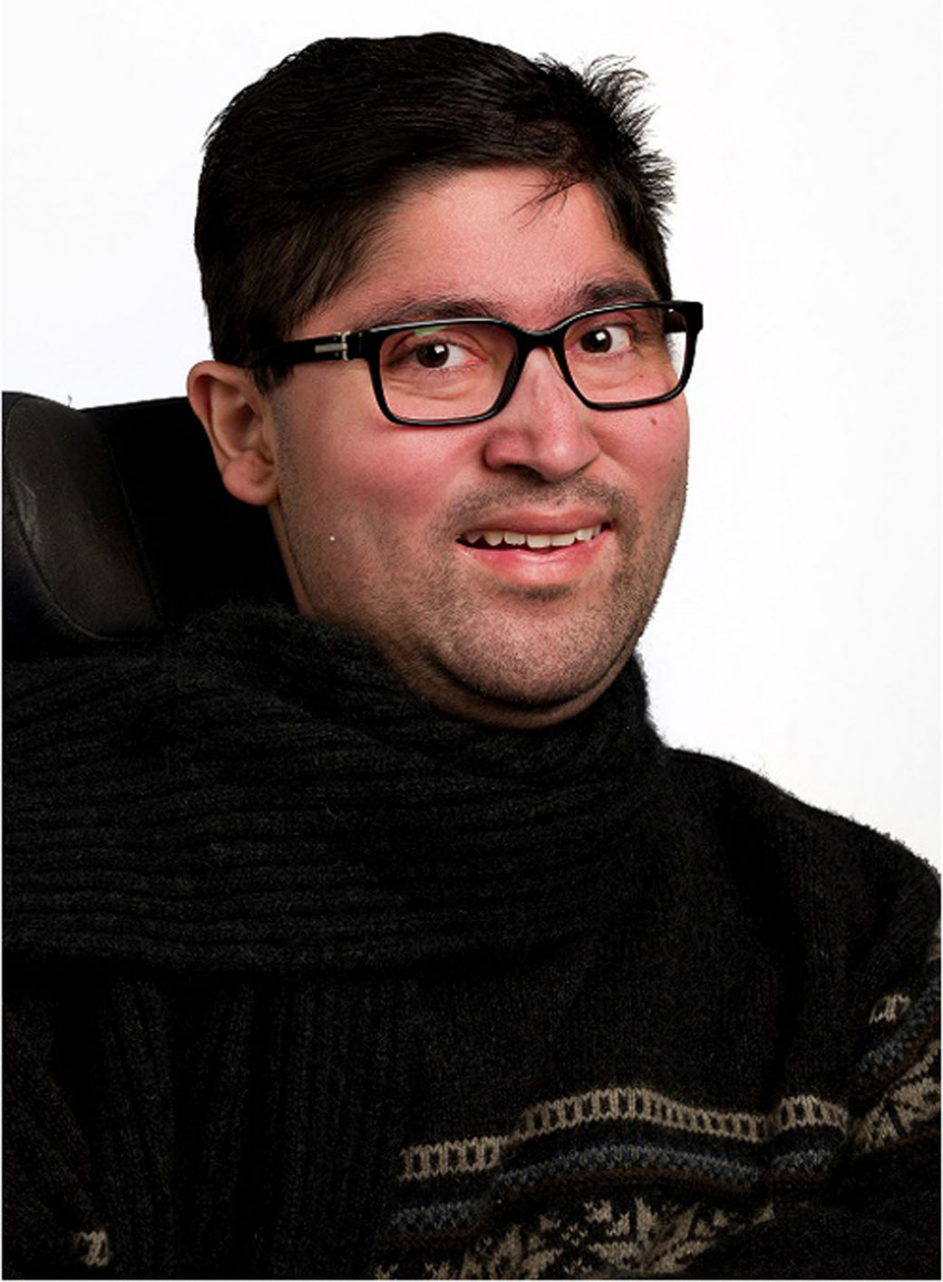


**Savant Singh Thakur [10 December 1991–16 June 2019]**

**What was Savant's scientific background and the general focus of your lab?**

Savant was a talented and determined research scholar, undertaking his Ph.D. in the Centre for Muscle Research in the Department of Physiology at The University of Melbourne. His scientific background was in the biomedical sciences, with a focus on physiology and cell biology.

The Centre studies the pathophysiology of muscle diseases and muscle wasting disorders, muscle injury and repair, and topics related to muscle growth and metabolism, relevant to applications in livestock production and aquaculture.

Savant was studying the role of heat shock proteins in muscle regeneration - a topic of direct relevance to understanding the muscle defects associated with Duchenne muscular dystrophy (DMD), the most severe of the muscular dystrophies. In fact, Savant had battled this terrible progressive disorder since childhood and he had been confined to a wheelchair even before his teens. His passion was to study DMD and hopefully identify something that would help or cure patients with this disorder. Support from The University of Melbourne allowed Savant to pursue his dream and he was an inspirational role model to all of us in science and biomedical research.

Savant was in the final stages of his candidature and preparing his doctoral thesis when sadly he fell ill, was hospitalised and died on June 16, 2019, as a result of complications arising from DMD.

**How would you explain the main findings of the paper to non-scientific family and friends?**

Heat shock proteins (HSPs) are molecular chaperones expressed during times of cellular stress that help proteins fold back to their original conformation and restore function. Since many muscle-wasting conditions involve inflammation, atrophy and weakness, increasing HSP expression in skeletal muscle may have therapeutic potential, especially to enhance the repair of muscles after injury. In this paper, we showed that increasing expression of a specific HSP (HSP70) in muscle cells, promoted their fusion during development of muscle fibres, identifying a potential mechanism for how this could be used to enhance muscle repair after damage.

**What are the potential implications of these results for your field of research?**

HSPs are essential for maintaining normal protein structure and function. Treatments that can coordinate the expression of different HSPs, could be a strategy to help confer protection to affected muscles in DMD and other muscle disorders, and would represent a major clinical advance. Such treatments would have the potential to delay progression of the dystrophic pathology, enabling patients to live longer and with a better quality of life until such time that a viable cure is identified.

**Figure:**
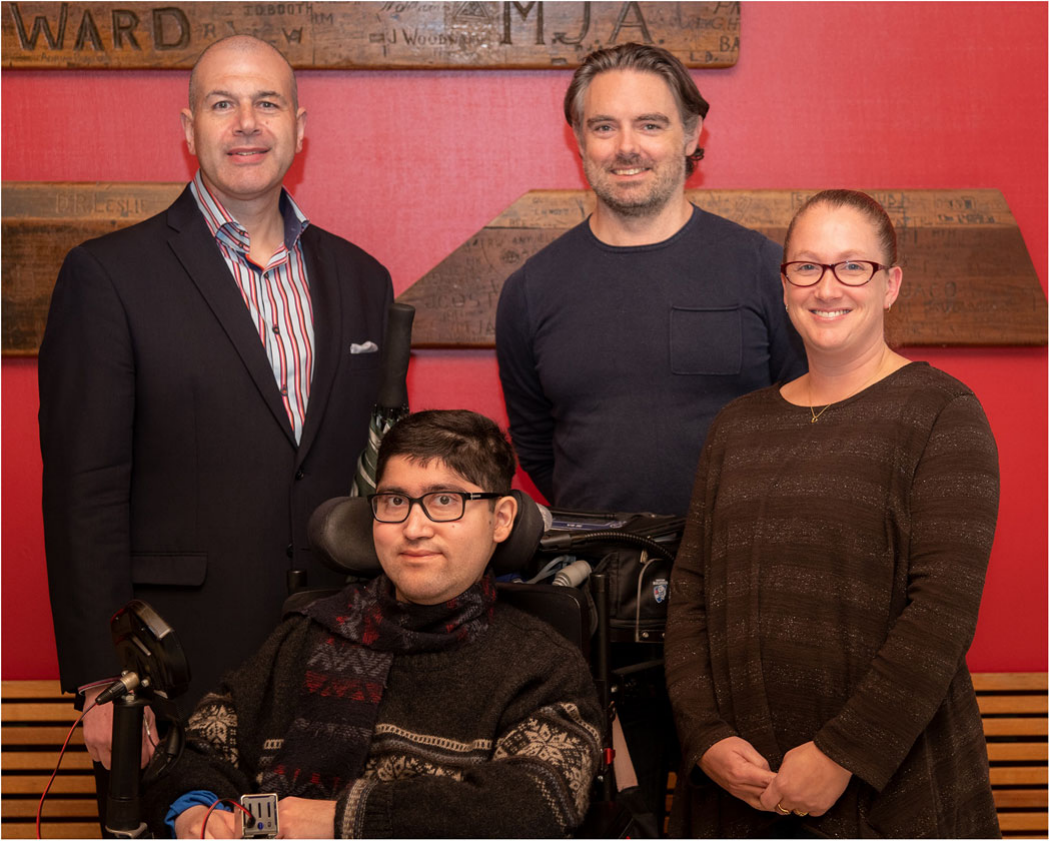
**Savant Thakur with his doctoral supervisors Prof. Gordon Lynch, Dr Kristy Swiderski and Dr James Ryall.**

**What would you want early-career scientists to take from Savant?**

Savant had a burning passion for science and strove hard to achieve his goals. He dealt with his condition with a quiet dignity and optimism, never once bemoaning his situation, despite having every right to do so. He didn't complain about trivial things, but focused on living a life of purpose. He just got on with the task and inspired others, every single day. I think Savant and his achievements should be celebrated as an inspiration to other Ph.D. scholars and to all of us in science.
